# Genomic Diversity of the Rarely Observed Genotype of the *Mycobacterium tuberculosis* Central Asian (CAS) Lineage 3 from North Brazil

**DOI:** 10.3390/microorganisms11010132

**Published:** 2023-01-04

**Authors:** Emilyn Costa Conceição, Marília Lima da Conceição, Davi Josué Marcon, Johannes Loubser, Gabrielly Leite Andrade, Sandro Patroca da Silva, Ana Cecília Ribeiro Cruz, Abhinav Sharma, Philip Suffys, Karla Valéria Batista Lima

**Affiliations:** 1 Department of Science and Innovation—National Research Foundation Centre of Excellence for Biomedical Tuberculosis Research, South African Medical Research Council Centre for Tuberculosis Research, Division of Molecular Biology and Human Genetics, Faculty of Medicine and Health Sciences, Stellenbosch University, Tygerberg, Cape Town P.O. Box 241, South Africa; 2 Seção de Bacteriologia e Micologia, Instituto Evandro Chagas, Ananindeua 67030-000, PA, Brazil; 3 Centro de Genômica e Biologia de Sistemas, Universidade Federal do Pará, Belém 66075-110, PA, Brazil; 4 Departamento de Medicina, Centro Universitário do Pará—CESUPA, Belém 66613-903, PA, Brazil; 5 Seção de Arbovírus e Febres Hemorrágicas, Instituto Evandro Chagas, Ananindeua 67030-000, PA, Brazil; 6 Programa de Pós-graduação Biologia Parasitária na Amazônia, Universidade do Estado do Pará, Belém 66087-670, PA, Brazil; 7 Faculty of Engineering and Technology, Liverpool John Moores University (LJMU), Liverpool L35UX, UK; 8 Seqera Labs, 08005 Bacelona, Spain; 9 Laboratório de Biologia Molecular Aplicada a Micobactéria, Instituto Oswaldo Cruz, Fundação Oswaldo Cruz, Rio de Janeiro 21040-900, RJ, Brazil

**Keywords:** tuberculosis, *Mycobacterium tuberculosis*, Lineage 3, phylogenetic, whole-genome sequencing, Brazil

## Abstract

*Mycobacterium tuberculosis* (*Mtb*) Central Asian Strain (CAS) Lineage 3 (L3) genotype is predominantly found in East-Africa, Central-Asia, Western-Asia, and South-Asia; however, a new spoligotyping CAS/SIT2545 was found in northern regions of Brazil. We aimed to characterize and describe the genetic diversity and perform a phylogenetic assessment of this novel genotype. We performed 24-MIRU-VNTR loci and Whole-genome sequencing (WGS) of six Brazilian isolates previously spoligotyped. The libraries were prepared using a Nextera-XT kit and sequenced in a NextSeq 550 Illumina instrument. We performed lineage assignment and genomic characterization. From publicly available genomes of *Mtb* L3 and other lineages, we created a robust dataset to run the MTBSeq pipeline and perform a phylogenetic analysis. MIRU-VNTR and WGS confirmed CAS/SIT2545 belongs to L3. Out of 1691 genomes, 1350 (79.83%) passed in quality control (genomic coverage > 95%). Strain 431 differed in 52 single nucleotide variants (SNV), confirming it does not belong to the same transmission chain. The eight genomes from a global dataset clustered closer to Brazilian strains differed in >52 SNVs. We hypothesized L3 and L1 were introduced in Brazilian Northern in the same historical event; however, there is a need for additional studies exploring the genetic diversity of *Mtb* Brazilian Northern.

## 1. Introduction

*Mycobacterium tuberculosis* (*Mtb*) Lineage 3 (L3), associated with the Central Asian Strain (CAS) genotype, is predominantly found in East Africa, Central Asia, Western Asia, and South Asia. Strains with the CAS genotype are likely the ancestors of the Beijing family strains (Lineage 2) as they occur in the same geographical location [1,2].

Mtb L3 (CAS/Delhi) is classified as a “modern” lineage that is more virulent than the ancient lineages [3] and has the distinct characteristic of the TbD1 genome deletion. L3 is associated with major tuberculosis (TB) epidemics largely due to higher transmission rates compared to L1, L5, L6 and L7. Additionally, L3 is metabolically distinct from other lineages because it has an increased abundance of proteins involved in lipid metabolism, the ESX-3 secretion system, the TCA cycle and other metabolic pathways associated with increased fitness and survival capacity [4].

There is compelling evidence that CAS lineage may have spread across Africa and Eurasia through ancient African-Asian trade networks and the routes of the Silk Road early in the first millennium [1]. Molecular data and identifiers from the CAS strains deposited in SITIVIT2 largely originate from countries along this route: Afghanistan (3), Belgium (1), Bangladesh (8), Brazil (2), China (3), Ethiopia (7), Great Britain (3), India (100), Iran (4), Iraque (24), Italy (1), Jamaica (1), Myanmar (6), Mozambique (1), Malawi (1), Marrocos (2), The Netherlands (1), Nepal (1), Oman (25), Pakistan (38), Saudi-Arabia (38), South Africa (5), Sudan (4), Somalia (9), Tanzania (7), Uganda (3), United States (15), Yemen (2), and Zambia (4). As L3 is underrepresented in this database, important numbers from medium and high-TB-income countries might be missing, since 189 entries are from an unknown origin.

A large study of *Mtb* based on 2030 L3 genomes from the endemic TB region, excluding strains from low-burden regions, demonstrated that L3 does not contain distinct sublineages. It was therefore proposed that L3 has a good temporal sign with a most recent common ancestor (MRCA) dating between the 2nd and the 13th century AD. Additionally, the authors suggested that *M. tuberculosis* complex (MTBC) studies describing Lineage 1 (L1) and L3 are of great importance since they greatly affect humans yet remain relatively understudied even on the global setting [2].

Northern Brazil has a high incidence of TB, but only a limited number of studies describing the genetic diversity of *Mtb* has come from this region [5]. Despite this, L1, rarely observed in South America, was described and genetically characterized in this region and postulated to have been introduced during with the trans-atlantic slave trade events in Brazil from East-African countries such as Mozambique and Malawi [6,7,8].

It is worth noting that CAS SIT 2545 (octal 703460000000331) was also found in the State of Pará, Brazil and according to the SITVIT2 database, TB infection with strains of this genotype is rare and has been observed thus far only in this Amazon region in the North of Brazil [6].

One of the earlier genotyping methods known as spoligotyping has been shown to poorly distinguish between closely related strains, resulting in incorrect assumptions regarding strain relatedness, origin and evolution [9]. Additionally, it has no resolution in terms of homoplasy, which leads to further misassumptions. Therefore, we aimed to describe the genetic variability based on 24-MIRU-VNTR and whole-genome sequences (WGS) of a selection of strains from the State of Pará, North Brazil, with SIT 2545 and their phylogenomic assessment in conjunction with publicly available genomes of strains of L3.

## 2. Materials and Methods

### 2.1. Study Population

Among 980 *Mtb* strains isolated between 1998 and 2010 in the state of Pará/Brazil that have undergone genotyping by spoligotyping, six identified as SIT 2545 were selected for this study. In vitro culturing, drug susceptibility testing (DST), Phenol-Chloroform DNA extraction and spoligotyping were performed according to Conceição et al. [6].

### 2.2. Genotyping Based on MIRU-VNTR

MIRU-VNTR typing was performed by amplifying 24-loci [10]. The PCR reaction was performed using a QIAGEN kit (HotStarTaq DNA polymerase, QIAGEN, Hilden, Germany), and the PCR products were analyzed on the ABI3130 sequencer. Alleles were assigned numerical values according to the number of repeats using GeneMapper Software v3.7 (Applied Biosystems, Waltham, MA, USA). Presently observed genotypes were compared to those present in the MIRU-VNTRplus database (https://www.miru-vntrplus.org/MIRU/index.faces, accessed on 7 July 2022) [11] and used as the basis of a neighbour-joining tree.

### 2.3. Whole-Genome Sequencing

DNA quantity for each sample was determined using Qubit dsDNA BR (Broad Range) assay kits (Thermo Fisher Scientific, Waltham, MA, USA). The DNA integrity was verified by gel electrophoresis on 1% ultrapure agarose gel in 1xTBE and using a 1 kb Plus DNA ladder (Thermo Fisher Scientific).

The libraries were prepared using the Nextera XT DNA Library Preparation Kit (Illumina, CA, USA) according to the manufacturer’s instructions and quality controlled using the Agilent High Sensitivity DNA Kit (Agilent, CA, USA), followed by the sequencing on an Illumina NextSeq 550 instrument using a 2 × 150 paired-end chemistry. The mean read depth was >20×.

### 2.4. Genome Analysis and Phylogenetic Reconstruction

The six raw data reads belonging to SIT 2545 and the robust dataset totalling 1691 *Mtb* genomes were subjected to quality controlled using FastQC v0.11.7 [12] before and after trimming for removal of adapter sequences and low-quality reads using Trimmomatic v0.33 [13] (parameters: LEADING:3 TRAILING:3 SLIDINGWINDOW:4:20 MINLEN:36). The trimmed reads were mapped against the *Mtb* H37Rv reference genome (GenBank accession number: NC_000962.3) through the MTBseq (v1.0.3) pipeline using BWA-MEM v0.7.16. Samtools v1.9 [14] was used to convert the SAM files to BAM format and sort mapped sequences and BAM file quality was checked using Qualimap [15]; sambamba v0.6.8 was used to mark read duplicates [16].

The MTBseq (v1.0.3) performed individual ran sample analysis individually in separate environments, removing samples with genome coverage < 95% and joining the remaining samples for comparative analysis. Software dependencies were installed using the Conda package manager through the Bioconda channel [17]. The infrastructure required to run the analysis was provided by Instituto Evandro Chagas (PBS cluster with 80 x86 CPUs with 1TB RAM).

As part of the MTBseq (v1.0.3) pipeline, single nucleotide variants (SNVs), which includes single nucleotide polymorphisms (SNPs) and small insertions and deletion (*indels*) were called and filtered based on the following default pipeline criteria: mapping quality ≥ 50, base alignment quality ≥ 23 and ≤ 2000 reads covering each site. Most variants detected in PE/PPE and other hard-to-map regions were automatically excluded based on these criteria and according to default MTBseq pipeline parameters. The resulting variant tables were then screened according to the following parameters: minimum read depth of 10 to call a variant and 2000 maximum read depth. Variant functional annotation was performed with SnpEff (v4.3) [18], while mutations were manually confirmed from BAM files.

The similarity matrix was constructed using the cohort analysis step of the MTBseq pipeline. High-confidence variable sites detected in all isolates, including coding and non-coding SNPs, were concatenated to generate a multi-FASTA file to construct a maximum likelihood phylogeny of the isolates included in this analysis with IQ-TREE (v2.2.0) with 1000 bootstrap pseudo-replicates using an ultrafast and automatic model selection method. We included *Mtb* genomes of representative human-adapted Lineages (L1–L9) and the outgroup *Mycobacterium canettii* (GenBank accession number: SAMEA3905803). The tree was annotated using the web tool iTOL (v6.6) (https://itol.embl.de/, accessed on 20 November 2022) [19].

The classification of the genotypes to the lineage and sublineage level and characterization of mutations associated with drug resistance was performed using TB-Profiler v4.4.0 [20], Spotyping v2.1 [21] and RD-analyzer v1.01 [22] in the command line version using default settings. We also performed de novo assembly using SPAdes to assemble the reads. Thereafter, the genome annotation was established using Prokka v11.3.4 [23].

Flowchart summarizing the various aspects of this study can be found in Figure S1.

### 2.5. Ethical Statement

This study was approved by the Ethical Committee of the Evandro Chagas Institute, number 059750/2017, CAAE: 69248217.0.0000.0019.

## 3. Results

### 3.1. MIRU-VNTR Result

The characteristics of the six selected MTBC strains of the CAS lineage with SIT 2545 (octal code 703660000000331) are described in Table 1. The strains had been isolated from 50% of male and 50% of female patients between 2001 and 2010 and therefore span a 10-year period. Strain 431, isolated in 2001, presented a multidrug resistance (MDR) profile, while the others were drug-susceptible.

**Figure 1:**
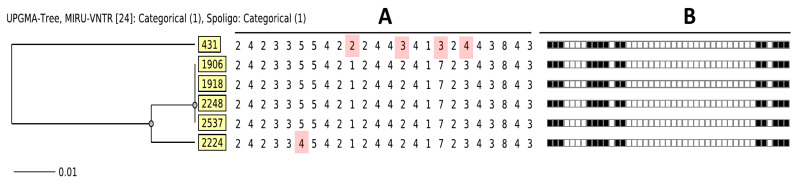
Phylogenetic tree (**A**) based on 24-MIRU-VNTR loci analysis on six *Mycobacterium tuberculosis* strains highlighting the quadruple-locus variation on strain 431 and a single-locus variation on strains 2224; identical spoligotyping profile (**B**) indicative of the Central-Asia (CAS) SIT 2545 genotype.

Upon comparison of the MIRU pattern(s) with the MIRU-VNTRplus database, the six isolates were grouped into a monophyletic branch related to the clade of Delhi/CAS. The 24-MIRU-VNTR loci pattern was identical for 19 loci, with a difference in two alleles in five loci (960_MIRU10, 2163b_QUB11b, 2461_ETRB, 2996_MIRU26, 3171_Mtub34) poorly discriminant with a discriminatory index of *h* = 0.13. The six strains were divided into two main groups: (1) orphan profile strain 431 and (2) a cluster harboring strains 1906, 1918, 2248, and 2537. Strain 2224, also clusters with these strains, but with a single locus variation (SLV) (Figure 1).

### 3.2. Whole Genome Sequencing Analysis and Phylogenomic Assessment

On average, the WGS of each of each of the six strains had of 16.18 million pared reads per sample before trimming and 6.5 million reads after quality trimming at an average genome coverage of 254.54×, and 65.51% GC content. Individual genomic characteristics are described in Table 2 and Table S1.

**Figure 2:**
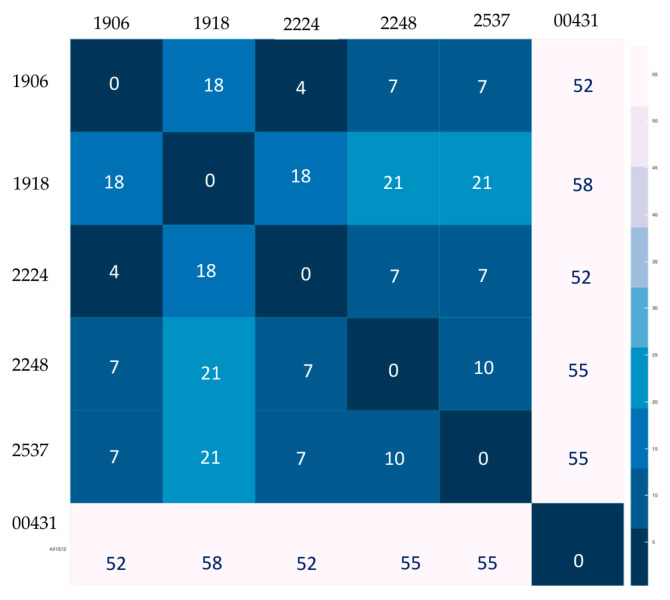
Heatmap based on single-nucleotide variant (SNV) distance among the six *Mycobacterium tuberculosis* Central-Asia (CAS) SIT 2545 genomes from Brazil. Darker blue is indicative of shorter genetic distance whereas light blue to white indicates a larger number of SNVs between isolates.

For the pairwise genetic comparisons based on SNVs, strains 1906, 1918, 2224, 2248 and 2537 differed by between 4 and 21 SNVs. Strain 431 was the outlier with at least 52 SNVs between the closest strain, 1906 (Figure 2).

**Figure 3:**
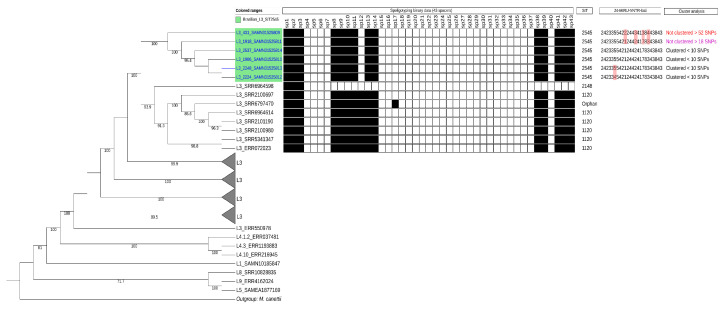
Cladogram based on the phylogenetic reconstruction of single-nucleotide variants (SNV) determined by whole-genome sequencing analysis with the MTBseq pipeline. The four collapsed clades represent 1431 of the including 1350 *Mycobacterium tuberculosis* L3 genomes acquired from publicly available databases, an outgroup *Mycobacterium canettii* and other *M. tuberculosis* representative lineages human-adapted (L1–L9). The six L3 genomes from Brazil (green) at the nodes of the cladogram is annotated with their spoligotyping pattern, MIRU-VNTR and 12 SNV pairwise genetic distance comparisons. The L3 genomes of the closely related subclade has a different spoligotyping pattern than Shared International Type (SIT) 2545.

Out of the 1691 genomes, 1350 (79.83%) passed the quality control (genomic coverage > 95%). The six novel strains, clustering together, were positioned on a subclade which shares a common ancestor with the second subclade with seven known L3 strains at the tips of the nodes. These genomes are from the United Kingdom (*n* = 4; ERR072023; SRR2100697; SRR2101190; and SRR2100980), and an unknown origin but possibly from Azerbaijan, Bangladesh, Belarus, Pakistan, Philippines, South Africa, and Ukraine (= 3; SRR6964598; SRR6797470; and SRR6964614). This distinct L3 clade comprising of two subclades were positioned the closest to four other L3 clades (Figure 3).

### 3.3. In Silico Genomic Analyses

RD-Analyzer, utilizing Region of Deletion (RD 750) and in silico spoligotyping also predicted the six isolates to be L3 strains (data not shown) as per the initial octal code (703460000000331) and distinct L3 spoligotyping pattern (Figure 1 and Figure 3). Additionally, TBProfiler predicted five of the strains (2248, 2224, 1906, 2537) to be MDR, similar to the initial phenotypic tests. Strain 431 was classified as isoniazid and streptomycin-resistant based on the presence of katG_p.Ser315Asn and gid_c.115_115del variants.

## 4. Discussion

Intra- and inter-CAS lineage genetic diversity could be attributed to historical factors which include, but is not limited to, human migrations, colonization and trade [4]. Diversity within and between CAS lineages could be explained by historical factors, human migrations, and trade [4]. The SIT 2545 strain dissemination in the most populated region in the Amazon region could have been due to the slave trade from East Africa that had a link with ancient and recent human history in the respective areas, reflecting the co-evolution of CAS lineage and host in a new geographic region. The CAS lineage in this scenario may reflect an introduction and adaptation of the particular *Mtb* strain to the population from North Brazil.

When compared to the more prevalent East-African-Indian (EAI) (L1) in Southeast Asia, India and East Africa, infection with CAS strains appears to be associated with younger patients (aged between 0 and 20 years) and MDR/extensively drug-resistant TB (XDR)TB [1].

The lineage CAS-Delhi was identified in two isolates in Brazil before, classified as newly arrived genotypes in Brazil that were likely under evolutive pressure resulting in subsequent loss of some spacers. This resulted in novel spoligotype patterns of lineages with well-documented specificity for distant continents due to being undocumented in the country/continent of origin of the lineage but exclusively observed in Brazil [24]. These isolates were detected as CAS lineage based on the spoligotyping method. However, possible convergent evolution resulted in the misclassification of these strains, and it was therefore impossible to confirm that these strains are related to the CAS-Delhi lineage commonly found in Asia. The lineage CAS-Delhi was reported using MIRU-VNTR 24 loci in four isolates from pulmonary TB patients in Minas Gerais, Brazil [25].

Due to spoligotyping not being able to accurately infer the true phylogenetic relationships owing to genetic convergence, we performed WGS to determine the true CAS lineage evolution in South America, in particular Northern Brazil. The 24-loci MIRU-VNTR was performed to provide a preliminary overview of the global population structure and spatial distribution of L3 strains found in the region. WGS further revealed the global phylogeny, geographically differentiated pattern, and related clusters.

We provide new evidence that L3 is found in Brazil, which therefore strongly suggests that an introductory event of the CAS-Delhi lineage might have occurred historically on the South American continent. This correlates with a previous study where two lineages CAS-Delhi (L3) were found in Ecuador, a region in which L4 is the most prevalent (98.4%). In this study, 24-MIRU-VNTR loci were used as the genotyping method in this first non-Brazilian report of CAS-Delhi in South America [26].

Here, based on MIRU-VNTR applied for CAS-Delhi strains, nine VNTR loci (4052-QUB26, 2996-MIRU26, 424-Mtub04, 960-MIRU10, 4156-QUB4156c, 3192-MIRU31, 2165-ETRA, 4348-MIRU39, and 2059-MIRU20) exhibited high discrimination [27], which is similar to the five most discriminatory loci in our study (960-MIRU10, 2163b-QUB11b, 2461-ETRB, 2996-MIRU26, 3171-Mtub34).

The *Mtb* L3 is not the only lineage with specific features likely to have adapted upon geographical introduction into Brazil. Similarly, L1, which commonly has a low correlation to transmissibility and virulence and is generally present in a restricted geographical distribution in Eastern Africa and the South of India, was also found in Pará and has shown different profiles as resistance to antibiotics being MDR [8]. This pattern of evolution was identified using the WGS method.

From our results of the six SIT 2545 strains, interesting observations were made when comparing 24-MIRU-VNTR loci and SNV-distance from WGS analysis. For example, strain 2224 differed by one MIRU locus from the other strains but had a mere a 4-SNV difference compared to strain 1906. In contrast, isolate 1918 appeared to be closely related to the other three strains based on MIRU-VNTR clustering analysis (Figure 1), but the SNV difference was at least 18 (Figure 2). This emphasizes the need to not only rely on more traditional genotyping methods, but also to utilize WGS analysis which provides a more accurate representation of strain relatedness.

Although the State of Pará has the fifth highest number of emerging TB cases in Brazil, molecular characterization of these strains is underutilized in this region. For comparison, MIRU-VNTR and WGS are used more widely in the Southeast, South, and Central West region [5]. The MDR profile of strain 431 (isolated in 2001) and the susceptible profiles of the other strains isolated after 2001 could imply that there are possibly several missed cases yet to be discovered, described and characterized with a similar SIT profile.

The divergence observed In MIRU-VNTR loci and WGS suggests that we are missing epidemiologically linked strains might be a limitation of our study, but it does suggest that there might be more clusters and outbreaks caused by this strain that was missed. Additionally, comparative analysis with the MTBseq pipeline requires excellent coverage of the genomes of interest, and 341 (20.16%) of the genomes downloaded from public databases.

Gold standard genotyping methods are often overlooked in regions with only a small number of laboratories, professionals, and appropriate supplies. Subsequently, the accurate identification and classification of *Mtb* is delayed, which leads to multiple health risk issues. These include increased community exposure to the bacillus, difficulties in outbreak investigations, transmission dynamics and ultimately, rapid access to an effective treatment. Large-scale genotyping is also needed to get better insights into the biological diversity and the evolution of the pathogen [28].

In a recent study it was demonstrated that the proportion of MDR and extensively drug-resistant (XDR) TB among CAS lineage is significantly higher than EAI lineage (from the eastern hemisphere and found in the Americas) [1], which highlights the need for an association of DST profile and genotypes applied to TB surveillance.

To date, the state of Pará has used WGS successfully to investigate the transmission of TB applied to *Mtb* L1 [2,7] and *Mycobacterium bovis* [29], albeit retrospectively. There is a necessity for WGS to be utilized even more widely and to expand its usage TB strains. This will greatly contribute to our enhanced understanding of the dynamics of TB transmission in the local population and pathogen-host co-evolution in Northern Brazil.

Genotyping of MTBC strains is the basis of establishing the phylogeny of this complex and complements TB transmission studies. The latter provides important guidelines for health agencies responsible for screening and interventions to implement strategies for the prevention and treatment of the disease and identify areas of higher priority.

## 5. Conclusions

This is a pioneer study confirming the presence of *Mtb* L3 in Brazil through 24-MIRU-VNTR and WGS. The comparison against an international dataset including 1350 genomes confirmed SIT 2545 belongs to a separate cluster. The evaluation of the transmissibility and the search for the possible origins of this lineage in the country based on global phylogenomic have great relevance for the maintenance of continued surveillance and for the promotion of public health. Our study contributes to further understanding of the *Mtb* genetic diversity in a high-burden TB area and pinpoints the need to improve genetic/genomic surveillance of the *Mtb* population and unravel the evolution and diversity of L3 in Brazil and South America.

## Figures and Tables

**Table 1:** Strain isolation year, drug-susceptibility test and sociodemographic characteristics associated with the mycobacterial interspersed repetitive unit—variable number of tandem repeats (MIRU-VNTR) and whole-genome sequencing (WGS) clustering analysis.

Strain	Year	DST ^1^	Sex	Age	MIRU-VNTR ^2^	WGS Cluster ^3^
431	2001	MDR	F	34	242335542**2**24434138**4**43843	Non clustered
1906	2005	Susc	M	29	242335542124424178343843	Cluster 1
1918	2010	Susc	F	61	242335542124424178343843	Non clustered
2224	2006	Susc	M	26	24233**4**542124424178343843	Cluster 1
2248	2006	Susc	M	-	242335542124424178343843	Cluster 1
2537	2008	Susc	F	38	242335542124424178343843	Cluster 1

^1^ Drug susceptibility test based on Proportion Method: multidrug-resistant (MDR) tuberculosis, simultaneous resistance to rifampicin and isoniazid, drug-susceptible (Susc). ^2^ Mycobacterial interspersed repetitive unit—variable number of tandem repeats (MIRU-VNTR) profile arranged in a crescent order of the 24-loci. ^3^ Whole-genome sequencing (WGS) cluster interpretation based on 12 SNPs cut-off. Bold/underline: it highlights the alleles differences from the majority of cohort result.

**Table 2:** Core genomic characteristics of the six *Mycobacterium tuberculosis* Lineage 3 strains from Northern Brazil based on the outputs of the reference-based MTBseq pipeline, Prokka annotation and de novo assembly analysis.

Strain	Mean Coverage	Mapping ^1^	Contigs	Largest Contig	N_50_	N_75_	Insertion ^1^	Deletion ^1^	SNPs ^1^
431	261.13	99.26%	165	173,577	61,250	39,904	109	162	1395
1906	277.38	99.23%	167	155,704	60,542	37,278	118	130	1381
1918	281.59	99.29%	162	161,736	62,665	37,278	139	120	1402
2224	267.62	99.32%	158	158,538	55,650	35,572	131	135	1387
2248	187.94	99.30%	178	214,707	52,142	33,124	135	121	1369
2537	251.62	99.33%	164	179,030	56,137	35,288	127	120	1386

^1^ Against the *Mycobacterium tuberculosis* reference H37Rv (NC_000962.3).

## Data Availability

The six raw sequence reads were deposited at NCBI under the Bioproject accession ID PRJNA895862.

## Supplementary Materials

The following supporting information can be downloaded at: https://www.mdpi.com/article/10.3390/microorganisms11010132/s1, Table S1: Statistics generated by MTBSeq pipeline of the *1350 M. tuberculosis* L3 genomes. Figure S1: Flowchart summarizing the various aspects of this study.
