# Supplementary figures and images for: Genomic Diversity of the Rarely Observed Genotype of the Mycobacterium tuberculosis Central Asian (CAS) Lineage 3 from North Brazil
Source: Microorganisms. 2023 Jan 4;11(1):132. doi: 10.3390/microorganisms11010132 (PMC9862801; doi:10.3390/microorganisms11010132)

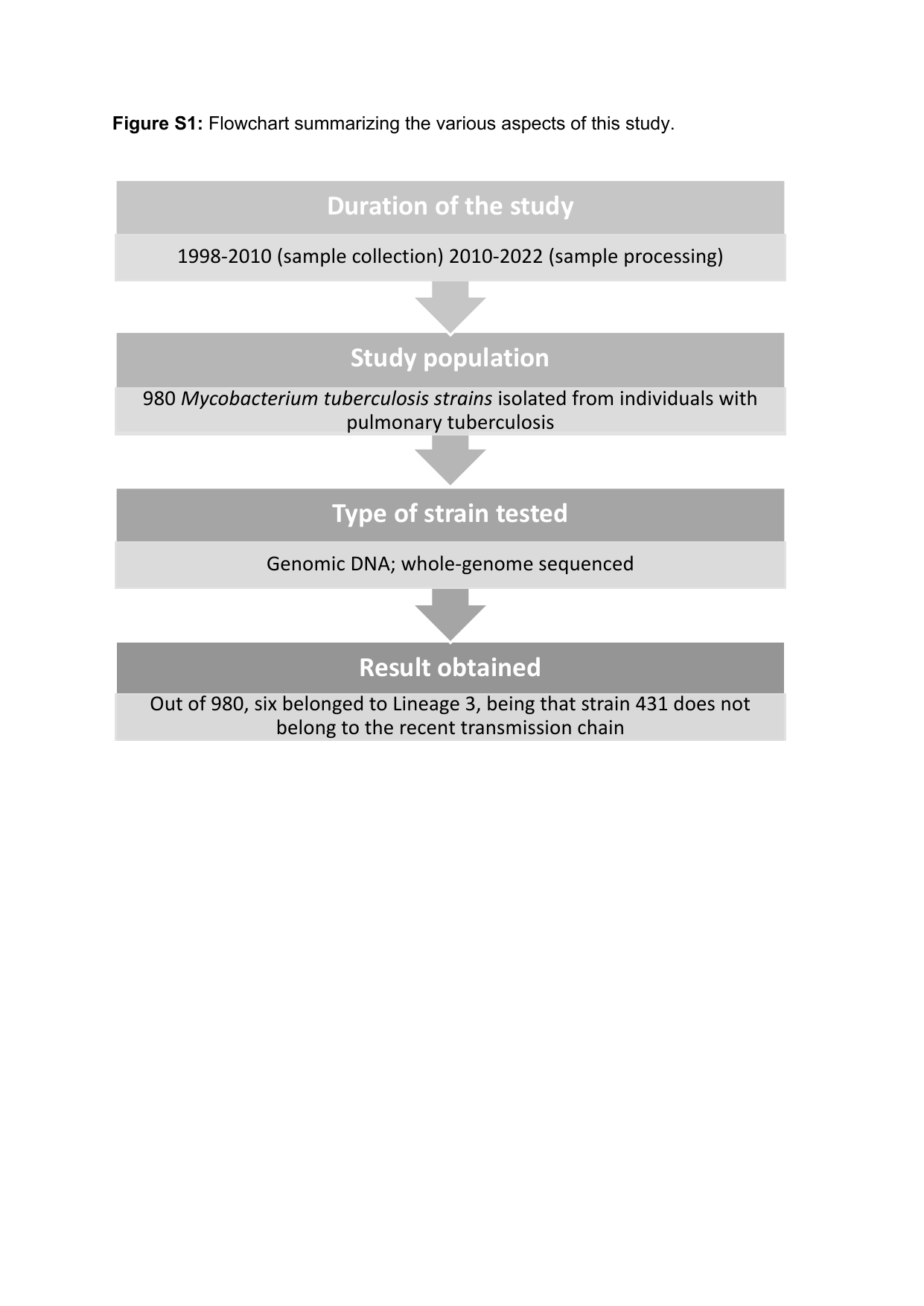

Supplement: Supplementary file 1 [file microorganisms-11-00132-s001.zip › SupFile/Figure_S1.tiff]
